# Supplementary figures and images for: Evolution of a functionally intact but antigenically distinct DENV fusion loop
Source: eLife. 2023 Sep 19;12:RP87555. doi: 10.7554/eLife.87555 (PMC10508882; doi:10.7554/eLife.87555)

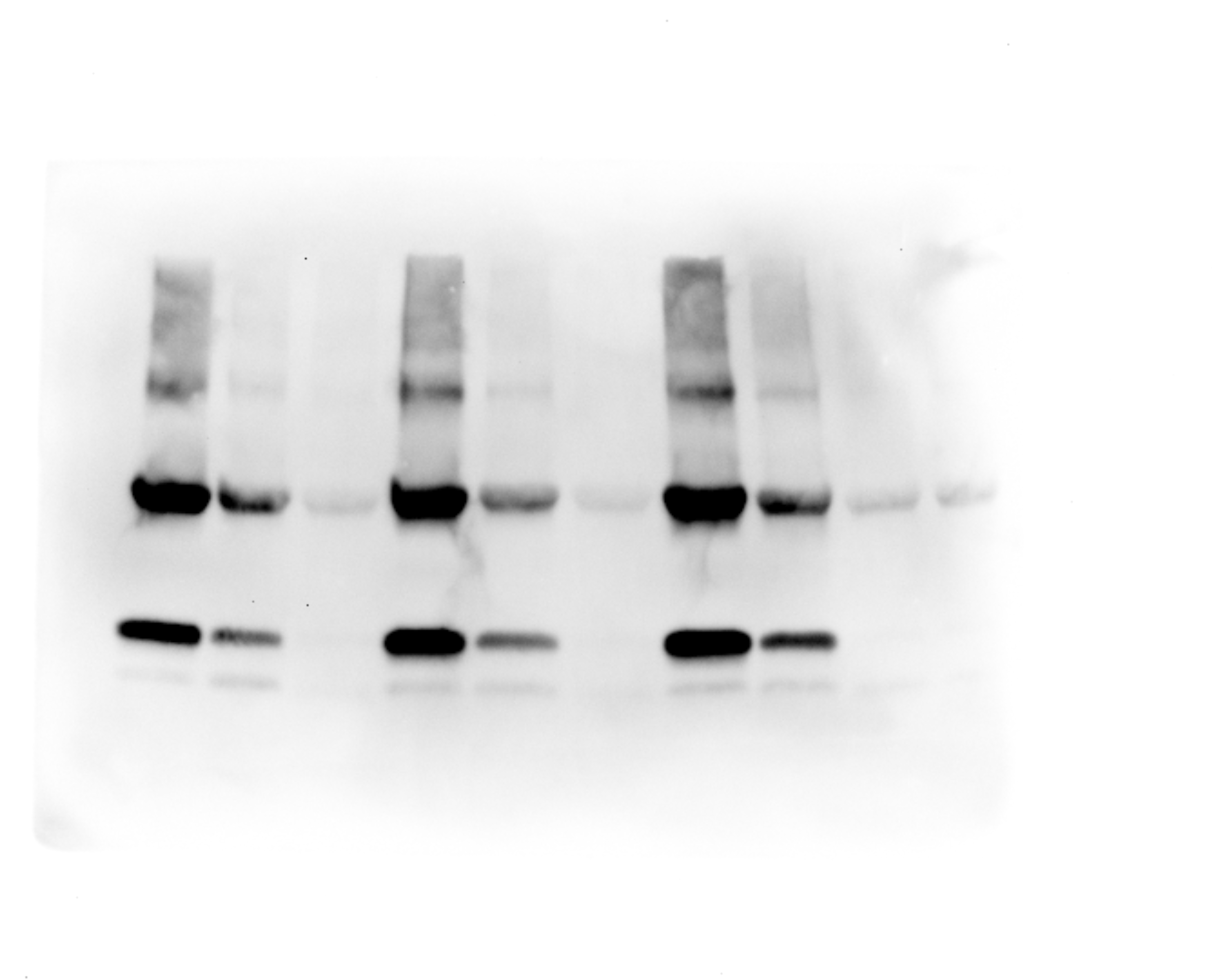

Supplement: Figure 2—source data 1. [file elife-87555-fig2-data1.zip › Figure 2-source data 1/02_20220407_181148_CHEMIChemi.tif]

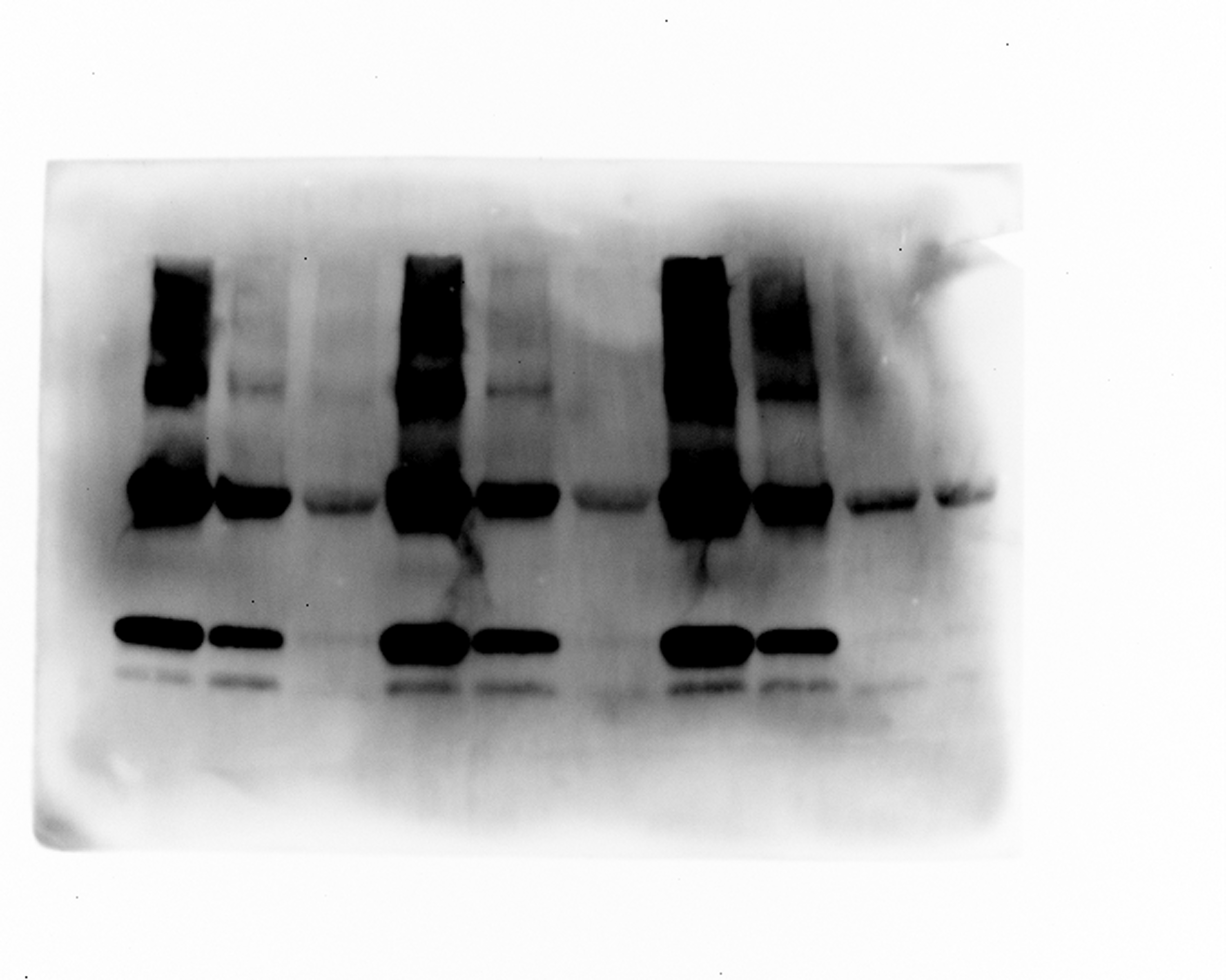

Supplement: Figure 2—source data 1. [file elife-87555-fig2-data1.zip › Figure 2-source data 1/03_20220407_181216_CHEMI.tif]

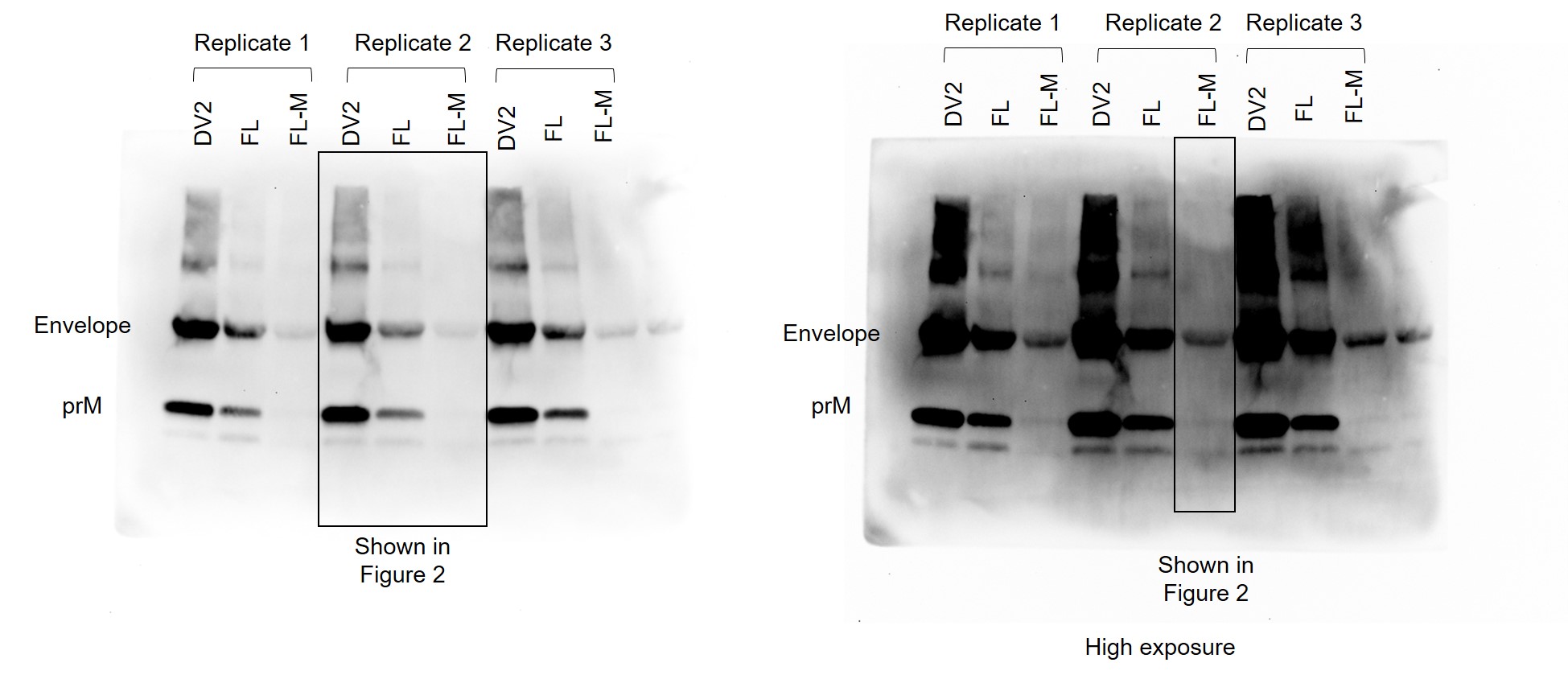

Supplement: Figure 2—source data 1. [file elife-87555-fig2-data1.zip › Figure 2-source data 1/Figure 2 gels.jpg]
